# Assessing a Method of Mechanical Cervical Dislocation as a Humane Option for On-Farm Killing Using Anesthetized Poults and Young Turkeys

**DOI:** 10.3389/fvets.2018.00275

**Published:** 2018-11-07

**Authors:** Caitlin R. Woolcott, Stephanie Torrey, Patricia V. Turner, Heather Chalmers, Lena J. Levison, Karen Schwean-Lardner, Tina M. Widowski

**Affiliations:** ^1^Department of Animal Biosciences, University of Guelph, Guelph, ON, Canada; ^2^Department of Pathobiology, University of Guelph, Guelph, ON, Canada; ^3^Ontario Veterinary College Health Science Centre, University of Guelph, Guelph, ON, Canada; ^4^Animal Care Services, University of Guelph, Guelph, ON, Canada; ^5^Department of Animal and Poultry Science, University of Saskatchewan, Saskatoon, SK, Canada

**Keywords:** turkey, on-farm killing, cervical dislocation, animal welfare, reflexes, insensibility

## Abstract

Our objective was to determine the efficacy of manual cervical dislocation vs. a mechanical cervical dislocation device for on-farm killing of poults and young turkeys. Forty-two 1- and 3-week old turkeys were randomly assigned to one of three experimental groups: awake manual cervical dislocation (CD), anesthetized manual cervical dislocation (aCD), or anesthetized mechanical cervical dislocation (MCD). Anesthetized birds received an intramuscular dose of 0.3 mg/kg medetomidine and 30 mg/kg of ketamine to achieve a light plane of anesthesia. A comparison of CD vs. aCD responses indicated that the anesthetic plane did not affect jaw tone or pupillary light reflex, indicators of loss of sensibility and brain death, respectively. MCD was unsuccessful for killing 1-week old poults as indicated by the ongoing presence of the pupillary eye reflex as well as failure to achieve cardiac arrest within 5 min in 5 of 5 birds. Radiographs also indicated no vertebral dislocation or fracture. Pupillary light reflex was present in 98% and jaw tone was present in 73% of turkeys, respectively, for all groups combined, but retention of the pupillary light reflex (*P* < 0.001) and jaw tone (*P* = 0.001) was longer for birds killed by MCD. Time to last movement (*P* = 0.797) and cardiac arrest (*P* = 0.057) did not differ between method. Survey radiographs demonstrated an effect of method for the average displacement distance at the site of vertebral dislocation, with a greater distance observed in birds killed by CD compared to MCD (*P* = 0.003). A method by age interaction was observed between CD and MCD for the number of birds with fractures; more vertebral fractures were observed in 3-week old turkeys killed with MCD compared to CD (*P* = 0.047). Upon gross examination, the majority of birds killed by either method had minimal to no hemorrhage within the brain and spinal cord. However, turkeys killed using CD had more microscopic subdural brain hemorrhage (*P* = 0.020). Ante-mortem and post-mortem measures suggest that neither manual CD nor the MCD tool used in this study caused immediate insensibility, but CD resulted in a shorter latency to brain death and fewer fractures compared to MCD.

## Introduction

The first few weeks post-hatch are critical to the survivability of the turkey poult, with the greatest susceptibility to early poult mortality occurring between 4 and 9 days (1). Newly hatched poults can have developmental abnormalities that make them unable to walk, eat, and drink; these unthrifty poults may harbor pathogens that pose a risk to the rest of the flock. Therefore, early identification and immediate culling of compromised poults are recommended to improve animal welfare and to reduce the risk for disease (2). In a 2008 survey of Canadian turkey producers, 32% of respondents indicated that of all age classes of turkeys, poults required euthanasia most frequently; in addition, 22% of participants identified the early grow-out stage as requiring euthanasia most frequently (3). Despite the need for humane killing methods for these early age classes, no studies have specifically investigated methods for euthanasia for poults or young turkeys.

In Canada, methods of euthanasia that are acceptable with conditions for poults and young turkeys <3 kg include gas inhalation, manual, and mechanical cervical dislocation, decapitation, blunt force trauma, and captive bolt devices (2). Cervical dislocation is acceptable if it results in luxation of the cervical vertebrae without crushing of the vertebrae or spinal cord (4). Cervical dislocation requires little to no equipment and stock people can be readily trained on proper technique. Because of this, cervical dislocation is the most common method used for the on-farm killing of poultry (3–5). When performed correctly, cervical dislocation separates the cervical vertebrae, ideally between the skull (C0), and the first cervical vertebrae (C1), completely transecting the spinal cord and disrupting blood vessels. Death is thought to be caused by cerebral ischemia and damage to the brainstem and spinal cord (6).

Cervical dislocation can be applied manually or mechanically (with the aid of a tool). For manual cervical dislocation, the size of the bird can affect technique and efficacy as heavier, adult turkeys may be too large and heavily muscled to properly restrain and to dislocate the neck. Conversely, young birds may be too small to use proper hand placement. Improper cervical dislocation may result in incomplete vertebral separation, dislocation lower in the vertebral column and failure to completely transect the spinal cord. Manual cervical dislocation of small birds can result in decapitation, which is acceptable for animal welfare but can be aesthetically distressing to the stock person and may pose a disease risk. Therefore, producers have begun to explore the use of mechanical cervical dislocation (MCD) to address issues related to bird size. A scissor-like mechanical cervical dislocation device (the Koechner Euthanizing Device, KED) is now commercially available for poultry euthanasia (7). A sequela to mechanical cervical dislocation is the risk of vertebral crushing. According to AVMA guidelines (4), vertebral crushing must not occur unless the bird is first rendered insensible. A few variations of mechanical or assisted cervical dislocation techniques exist for chicks and poults whereby dislocation of the cervical vertebrae is achieved by pressing the neck of the bird against the corner of a table or by using the blunt edge of hemostats or scissors; however, little research has been carried out for these methods.

For a method of killing to be considered humane, pain and distress should be minimized and insensibility should be immediate or rapid, followed by a loss of respiratory function, and cardiac arrest (4). Therefore, determining the efficacy of physical killing methods for poultry often relies on clinical behavioral measures of sensibility (brainstem and spinal reflexes) followed by post-mortem evaluation of fatal cerebrospinal injuries. However, the evaluation of new killing techniques or tools on awake animals raises ethical concerns. Initial trials of novel tools or techniques are often conducted on cadavers [for example, layers, and broilers, (8)] with the killing potential assessed by evaluating damage to key anatomic structures. Preliminary tests for euthanasia methods have also been conducted on anesthetized animals [for example, piglets, (9)] to determine the capacity of the technique to abolish rhythmic breathing and induce cardiac arrest. The use of anesthetic agents allows for a loss of awareness and depression of pain sensation, thereby reducing distress that may be associated with the killing method. Brainstem and spinal reflexes can still be used to monitor the depth of anesthesia as well as loss of sensibility after stunning or during killing (10, 11) and these may vary depending on the level of anesthesia. During deep anesthesia in birds, voluntary blinking, palpebral reflex, corneal reflex, and pedal reflex are absent, but pupillary reflex is present (12). In turkeys anesthetized with sevoflurane, nictitating membrane and pupillary reflexes persisted to the levels of general anesthesia and deep hypnosis until brain death; the level of anesthesia was confirmed by EEG (11). Sandercock and colleagues (11) also showed that jaw tone was the distinguishing measure between an awake and an insensible bird. It should be noted that different agents used for anesthesia can affect various reflexes differently. For example, ketamine can influence muscle tone and may influence the duration of jaw tone (12). Ketamine is often used in combination with other drugs such as xylazine or medetomidine for avian anesthesia to provide muscle relaxation, analgesia, and sedation which smooths induction and recovery (13, 14).

The main objective of this study was to determine the efficacy of manual cervical dislocation (CD) vs. a mechanical cervical dislocation (MCD) device designed specifically for the on-farm killing of poults and young turkeys. For ethical reasons, the MCD was tested on anesthetized birds. Therefore, a secondary objective was to determine the effect of the anesthetic agents on ante-mortem measures commonly used for assessing killing methods in birds.

## Materials

All procedures used in this study were approved by the University of Guelph Animal Care Committee (AUP #3321). The University of Guelph holds a Good Animal Practice certificate issued by the Canadian Council on Animal Care and is registered under the Ontario Animals for Research Act. All turkeys enrolled in the study were identified by farm personnel as sick or injured and requiring euthanasia based on the farm animal care protocol.

### Euthanasia method

**Figure 1:**
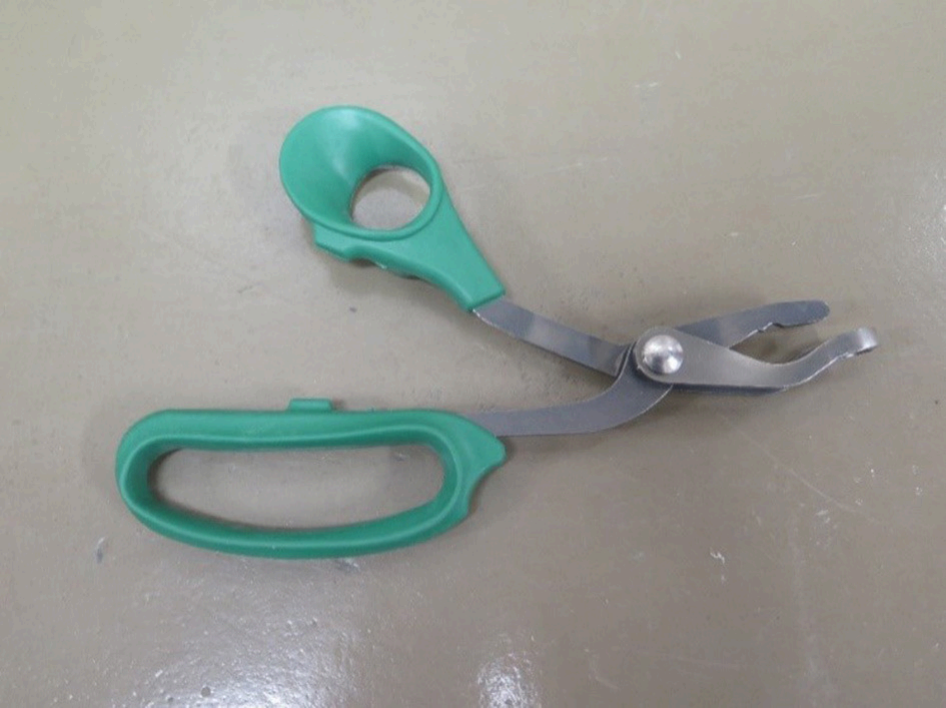
Koechner device. Single angle (top) blade to be placed above the top of the neck at the base of the skull. Double angle (bottom) blade to be placed under the back end of the bird's jaw.

The Koechner Euthanizing Device or KED (U.S. Patent Number 8,152,605) is a mechanical cervical dislocation device created by Koechner MFG. Co., Inc (7). Four variations of the KED exist for different weight classes of birds: KED-small (Model S for use on chicks and poults), KED-medium (Model C consisting of a 69 cm handle with gear for better leverage), KED-large (Model T consisting of a 102 cm with gear for use on large turkeys), and KED-xl (Model B composed of a 102 cm handle for use primarily on large turkeys and heavy breeders) (7). The KED-s, recommended for birds up to 1.8 kg, was used for this study (Figure 1).

### Anesthesia and euthanasia procedure

Forty-two turkeys, ~1- and 3-weeks of age and ranging from 44 to 586 g were enrolled (Table 1). Birds were randomly selected from the holding crate and assigned to one of three euthanasia groups on the day of each trial: awake manual cervical dislocation (CD), anesthetized manual cervical dislocation (aCD), or anesthetized MCD (aMCD). Three experienced stock people performed CD and one researcher performed MCD. Prior to the application on live birds, the device was used on 12 cadavers within the same weight ranges to determine placement and pressure required. All birds were manually restrained on a table in a sternal recumbent position.

**Table 1:** Distribution of treatment groups by age, weight, and killing method, with the sample size indicated for each.

**Age (wks)**	**Weight (g)**	Method^*^	**Sample size**
1	60 ± 6.3	Awake CD	5
	44 ± 4.0	Anesthetized CD	5
	56 ± 4.0	Anesthetized MCD	5
3	493 ± 64.7	Awake CD	9
	520 ± 64.8	Anesthetized CD	9
	566 ± 57.6	Anesthetized MCD	9

* *Methods of killing include manual cervical dislocation (CD) and mechanical cervical dislocation (MCD)*.

A cocktail of medetomidine injectable (Cepetor™, DIN: 02337177, Modern Veterinary Therapeutics, LLC, Miami, FL, USA) 1 mg/ml and ketamine (Ketaset®, DIN: 02173239, Pfizer Animal Health, Kirkland, QC, CAN) 100 mg/ml was prepared, respectively, resulting in a final intramuscular injected dose of 0.3 mg/kg medetomidine and 30 mg/kg of ketamine. This dosage was determined based on the results of a pilot project evaluating several drug and dose combinations in chickens (data not shown). The chosen drug combination can result in light to medium surgical anesthesia in birds (15). Only one dose was required to achieve the desired level of deep sedation in all turkeys. Following injections, birds were placed in crates in a dark room for 15 min. Birds were assessed prior to the start of the procedure and found to be unresponsive to general handling, with minimal or absent withdrawal response when the toe was pinched. Turkeys were assessed to ensure that the pupillary reflex was present.

CD was applied to the 3-week-old poults according to the AVMA (4) guidelines, that is, the legs or wings were grasped and the head was pulled using a ventrodorsal rotational force. A modified technique was used for the 1-week-old poults, the body was held in one hand with the first finger and thumb bracing the neck below the desired dislocation site while the other hand applied a ventrodorsal rotational force to the head of the bird. MCD was applied according to the manufacturer's instructions with the double blade placed under the angle of the jaw of the bird, and the single blade above the top of the neck at the base of the skull (7). The handles were brought together quickly and firmly with the goal of causing dislocation, and then the device was removed from the neck of the bird.

### Assessment of reflex and behavioral measures

Immediately after either kill-method was applied, turkeys were assessed for the presence of a pupillary light reflex, nictitating membrane reflex, gasping reflex, and jaw tone. Reflexes were checked every 15 s until the onset of cardiac arrest. If loss of pupillary and/or nictitating membrane reflex was not achieved within a 5-min period after the mechanical cervical dislocation method was applied, the turkeys were euthanized with a secondary method (manual cervical dislocation). Detailed definitions of the ante-mortem measures collected are described elsewhere (16).

The onset and duration of involuntary movements (neuromuscular spasms) were recorded. Time to last movement was recorded using end time of clonic and tonic convulsions, whichever occurred last. The presence and duration of the heartbeat were monitored through palpation and indirect auscultation. Cardiac arrest was estimated when no heartbeat could be palpated or auscultated with a stethoscope. Time of death was estimated when all reflexes had ceased entirely and cardiac arrest had occurred. For reliability, a single observer unblinded to treatment recorded all measures for all trials.

### Assessment of cerebrospinal trauma

Following confirmation of death, turkeys were radiographed, dissected, and macroscopically scored at the University of Guelph Arkell Research Station.

**Figure 2:**
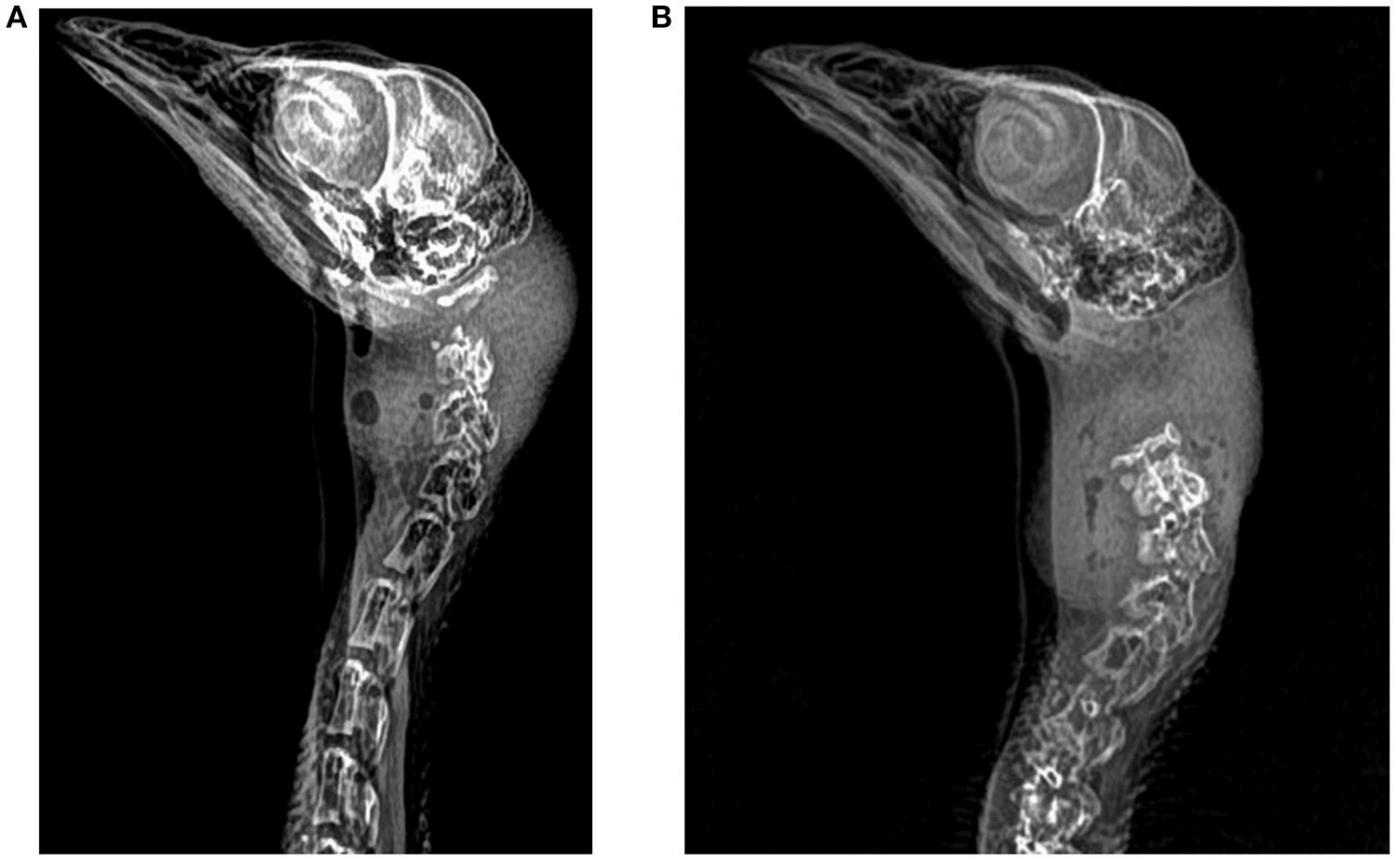
Three-week-old anesthetized turkeys. **(A)** Survey radiograph demonstrating dislocation and fracture of the C1 and C2 vertebrae in a young turkey euthanized by mechanical cervical dislocation **(B)** Survey radiograph demonstrating dislocation between C0 and C1 vertebrae in a young turkey euthanized by manual cervical dislocation.

Radiographs were taken for all turkeys and used to assess dislocation site, displacement distance, number of fractures, and fracture type. Four views were captured of each poult (dorsal, ventral, right lateral, left lateral) using a portable x-ray unit (Poskom VET-20BT, Deerfield, Illinois) with the following settings: 90 kVp, 20 mA power, and 0.4 to 20 mA output. The x-ray unit was held 82 cm away from the imaging receptor panel and the panel had the following parameters: 19.65 × 23.6 cm image matrix, a-SI TFT-PIN (thin filament transistors), 77-micron pixel pitch, 6.0 to 7.0 Ip/mm resolution. Turkeys were placed directly onto the imaging panel. Radiographs were scored by a veterinary radiologist, blinded to the age of the bird and treatment. Fragmentation was defined as the presence of a fracture with the origin of the fragment unknown (17). Transverse fractures were defined as a fracture perpendicular to the long axis of the bone (17). Comminuted fractures were defined as fractures with more than one fracture line present (17). Radiographs were scored using the eFilm diagnostic viewing workstation (Merge Healthcare, Chicago, Illinois) and the right lateral view (Figure 2) with displacement distance calculated using a digital measurement tool (Merge Healthcare, Chicago, Illinois). If the right lateral view was not visible, the left lateral view was scored.

Gross dissection and macroscopic scoring of the brain and spinal cord hemorrhage were conducted on all turkeys that were successfully euthanized. External hemorrhage and skin laceration were assessed to determine any skin trauma or hemorrhage at the site of cervical dislocation. Macroscopic scoring was conducted following the same procedure as Woolcott et al. (16). Subcutaneous hemorrhage was scored by excising the skin around the head and neck of the bird to measure the degree of hemorrhage. The calvarium (skull cap) was then removed to determine the extent of hemorrhage on the subdural dorsal and subdural ventral surface of the brain. The dorsal aspect of the spinal column was then removed, and the spinal cord was assessed for hemorrhage and transection.

Following macroscopic scoring, the brains and spinal cords from 23 birds (roughly 5 brains and spinal cords per method per age group) were selected for microscopic scoring and placed in 10% buffered formalin for a minimum of 10 days for hardening. All trimming was completed by one individual to ensure consistency. Three sections of the brain (cerebrum, midbrain, and cerebellum) and three sections of the spinal cord (at the level of the first, second, and third cervical vertebrae; C1, C2, and C3, respectively) were sampled. Tissue sections were embedded in paraffin wax, trimmed and stained with hematoxylin and eosin (Animal Health Laboratory, University of Guelph). Each section was then microscopically examined and scored by a veterinary pathologist blinded to the age of the bird and treatment. The percentage of microscopic subdural hemorrhage and parenchymal hemorrhage was determined using a 0 to 4 scale from Woolcott et al. (16). Microscopic hemorrhage scores from each brain region were compared. The overall degree of damage for microscopic subdural and parenchymal hemorrhage was determined by assigning the greatest score recorded in any of the three areas.

### Statistical analyses

All statistical analyses were performed using SAS 9.4 (SAS Institute Inc., Cary, NC). Two separate sets of statistical analyses were used. The first analysis was used to compare anesthetized manual cervical dislocation to awake manual cervical dislocation to determine the effects of the anesthesia on each outcome variable. If there was an effect of the anesthetic, only anesthetized manual cervical dislocation was statistically compared to the mechanical cervical dislocation. If no significant difference was found, indicating no effect of anesthesia, data from both groups of birds were pooled to compare cervical dislocation (anesthetized and control) to anesthetized mechanical cervical dislocation. Fisher's Exact test was used to test the null hypothesis that the proportion of birds presenting ante-mortem measures following treatment was independent of killing method. Frequency tables were used to determine the number of birds presenting with ante-mortem measures.

Mixed model analyses were used to test the fixed effects of the method, age, and their interaction on the duration of pupillary light reflex, nictitating membrane reflex, gasping reflex, jaw tone, convulsions, and time at cardiac arrest. For the mixed model analyses of variance of durations, all variables, except for cardiac arrest, were log transformed to normalize the data. Raw means and standard errors are presented in the results. Generalized linear mixed models were used to test the effects of the method, age, and their interaction on macroscopic and microscopic damage using a multinomial distribution. Odds ratios were used to compare differences in the levels of fixed effects. If the results of odds ratios showed any significant method by age interactions, then another analysis was run separately for each method. For the microscopic scores, the effect of brain section on hemorrhage scores was first tested, and then the data were combined to compare treatment differences. Statistical significance was set at *P* < 0.05 for all analyses.

## Results

### Assessment of reflex and behavioral measures

The incidence of the various ante-mortem measures is presented in Table 2. For turkeys exhibiting these measures, the mean durations are shown in Table 3. Bird weight did not influence any ante-mortem measures for any method (*P* > 0.05).

**Table 2:** Number of young turkeys presenting with various reflexes and involuntary behaviors following application of the killing methods.

**Reflex**	**Method**	**Age (wks)**		***P*****-Value**			
		**1 (*N* = 5)**	**3 (*N* = 9)**	**Anesthesia*^a^***	**Method ^b^**	**Age**	**Method x Age**
Pupillary	CD	5	9	0.178	0.348	0.357	0.348
	aCD	4	9				
	MCD	5	9				
Nictitating	CD	5	8	**0.005**	**0.029**	0.717	0.904
	aCD	2	4				
	MCD	4	8				
Gasping	CD	4	5	0.304	**0.018**	0.232	0.635
	aCD	3	3				
	MCD	5	8				
Jaw tone	CD	5	7	0.762	0.200	0.746	0.746
	aCD	4	8				
	MCD	5	9				
Clonic	CD	5	9	**0.001**	0.819	<**0.001**	**0.018**
	aCD	3	3				
	MCD	5	0				
Tonic	CD	0	5	0.131	0.141	0.141	0.141
	aCD	0	1				
	MCD	0	0				

a *The effect of anesthesia compares the changes noted in birds anesthetized and killed by manual cervical dislocation (aCD) vs. changes in awake birds killed by manual cervical dislocation (CD)*.

b *If the effect of anesthesia was non-significant, CD (anesthetized and awake combined) was compared to mechanical cervical dislocation (MCD); if the effect of anesthesia was significant only anesthetized CD was compared to MCD*.

*Bold indicates significant values*.

**Table 3:** Mean duration (± SE) of ante-mortem reflexes and behaviors in young turkeys for each euthanasia method (CD, aCD, and MCD) and age group (1 week, 3 weeks).

**Variable**	**Method**			**Age^1^**	***P*****-value**			
**Age (wks)**	**CD**	**aCD**	**MCD**		**Anesthesia^2^**	**Method^3^**	**Age**	**Method × Age**
**Pupillary light reflex**								
1	108 ± 5.6^a^	124 ± 24.0^a^	300 ± 0^b^^c^	175 ± 23.4	0.132	<**0.001**	<**0.001**	**0.045**
3	73 ± 9.5^a^	105 ± 15.6^a^	133 ± 16.9^b^^d^	104 ± 9.3				
Mean^4^	86 ± 7.8	111 ± 12.8	193 ± 24.6					
**Nictitating membrane reflex**								
1	69 ± 9.0	23 ± 7.5	218 ± 30.3	115 ± 27.4	0.170	**0.023**	0.619	0.958
3	47 ± 12.2	49 ± 16.6	86 ± 9.7	63 ± 8.0				
Mean	55 ± 8.6	40 ± 12.0	130 ± 21.7					
**Gasping**								
1	45 ± 10.6	135 ± 15.0	225 ± 19.6	143 ± 25.0	**0.020**	0.508	0.070	0.529
3	42 ± 12.9	20 ± 5.0	77 ± 12.2	55 ± 9.2				
Mean	43 ± 8.1	78 ± 26.7	134 ± 23.1					
**Jaw tone**								
1	51 ± 9.0	98 ± 28.4	252 ± 24.8	136 ± 27.0	0.774	<**0.001**	0.165	0.666
3	79 ± 20.1	43 ± 11.1	112 ± 12.5	79 ± 10.0				
Mean	68 ± 12.6	61 ± 13.6	162 ± 21.9					

*^1^ All methods combined at that age group*.

*^2^ The effect of anesthesia compares the changes noted in birds anesthetized and killed by manual cervical dislocation (aCD) vs changes in awake birds killed by manual cervical dislocation (CD)*.

*^3^ If the effect of anesthesia was non-significant, CD (anesthetized and awake combined) was compared to mechanical cervical dislocation (MCD); if the effect of anesthesia was significant only anesthetized CD was compared to MCD. Only data from birds presenting responses were used*.

a, b *Indicates differences observed within a row (method difference within age)*.

c, d *Indicates differences observed within a column (age difference within a method)*.

*^4^ All ages combined within that method*.

*Bold indicates significant values*.

#### Effects of anesthesia on measures from birds killed by manual cervical dislocation

The only ante-mortem measures affected by anesthesia were the frequencies of birds presenting with positive nictitating membrane reflex, clonic convulsions, and the duration of gasping. Fewer anesthetized birds presented with nictitating membrane reflex (CD 94% vs. aCD 42%; *P* = 0.005) and clonic convulsions (CD 100% vs. aCD 60%; *P* = 0.001) than awake birds. However, of the birds presenting with positive nictitating membrane reflex, the duration was not different (*P* = 0.170). Duration of gasping was longer for aCD treatment compared to CD (*P* = 0.020). Times to last movement (*P* = 0.408) and cardiac arrest (*P* = 0.191) were the same for birds in both treatments, with a mean of 123 ± 27.3 s and 264 ± 24.1 s for CD and 133 ± 22.1 s and 303 ± 26.2 s for aCD, respectively.

An interaction between age and anesthesia was observed for the number of birds exhibiting tonic convulsions with a higher incidence in 3-week vs. 1-week old turkeys (33 vs. 0%; *P* = 0.028). An age by anesthesia interaction was also observed for the duration of gasping, with a longer duration in 1-week vs. 3-week old turkeys (Table 3; *P* = 0.001).

#### Effects CD vs. MCD

The MCD method was unsuccessful for killing 1-week-old turkeys as indicated by the prolonged presence of the pupillary eye reflex and failure to achieve cardiac arrest in any of the birds at the five-minute endpoint. These birds were radiographed while under anesthesia and then subsequently killed by manual CD. Data are presented in Tables 2, 3.

More birds were observed with a positive nictitating membrane reflex following MCD vs. aCD (85 vs. 42%; *P* = 0.029) and there was no effect of age (*P* = 0.717) nor a method by age interaction (*P* = 0.904; Table 2). Clonic convulsions showed a method by age interaction with a greater number of convulsions observed in 1-week vs. 3-week old turkeys when killed using the MCD (100 vs. 0%; *P* = 0.018). There was no effect of the method, age, or a method by age interaction on the duration of gasping (*P* = 0.508, *P* = 0.070, and *P* = 0.529, respectively).

There was no effect of the method on the number of birds presenting with a positive pupillary light reflex (*P* = 0.348), jaw tone (*P* = 0.200), or tonic convulsions (*P* = 0.141) as most birds exhibited pupillary light reflex and jaw tone, and few birds exhibited tonic convulsions (Table 2). Similarly, no effect of age was observed for the presence of pupillary light reflex (*P* = 0.357), gasping (*P* = 0.232), jaw tone (*P* = 0.746), or tonic convulsions (*P* = 0.141). More birds were observed gasping following treatment with MCD compared to those killed by CD (93 vs. 54% for both ages combined; *P* = 0.018).

Duration of the nictitating membrane reflex was affected by method with birds killed by MCD demonstrating a longer duration compared to CD (*P* = 0.023). No effect of age (*P* = 0.619) or method by age interaction (*P* = 0.958) was observed for the duration of nictitating membrane reflex (Table 3). Duration of pupillary light reflex showed a method by age interaction in which 3-week old turkeys had a shorter duration of pupillary light reflex than 1-week old poults when using MCD (*P* = 0.045). An effect of the method was observed for the duration of jaw tone with birds killed by MCD demonstrating a longer duration compared to CD (*P* = 0.001). No effect of age (*P* = 0.165) or method by age interaction (*P* = 0.666) was observed.

### Post-mortem radiographic assessment

Results from the assessment of radiographs are shown in Table 4. No dislocation and no fractures were observed in any of the 1-week old poults following MCD. For the 1-week old poults killed with CD, the location of dislocation varied between the skull to C1, C1 to C2, and C2 to C3. For 3-week old turkeys killed with CD, the dislocation occurred between the skull and C1 in 56% of birds, and between C1 and C2 in 39% of birds. For 3-week old turkeys killed with MCD, the dislocation did not occur between the skull and C1 in any birds. Instead, the dislocation occurred between C1 and C2 in 44% of birds and between C2 and C3 in 66% of birds. At 3 weeks of age, one bird killed with CD and one bird killed with MCD had multiple dislocation sites.

**Table 4:** Results of radiographic scoring of awake and anesthetized turkeys killed by manual and mechanical cervical dislocation techniques.

**Age (wks)**	**Method**	**N**	**Dislocation site**^**1**^				**Average displacement distance (mm)**	**Number of birds with fractures**	**Fracture type**
			**Skull-C1**	**C1-C2**	**C2-C3**	**Indistinct**			
1	CD	5	1	1	2	1	4.3 ± 1.70 ^a^^c^	0	N/A
	aCD	5	1	3	0	1	3.8 ± 1.11 ^a^^c^	2	Transverse
	MCD	5	0	0	0	5	0.0 ^a^^d^	0^a^	N/A
3	CD^*^	9	5	4	1	0	6.7 ± 1.43^b^^c^	1^c^	Comminuted
	aCD	9	5	3	0	1	9.6 ± 1.94^b^^c^	3^c^	Comminuted
	MCD^*^	9	0	4	6	0	3.2 ± 0.64^b^^d^	7^bd^	Transverse, comminuted, fragmentation

*^1^Number of birds presenting dislocations at each site*.

* One bird killed with manual cervical dislocation (CD) and one bird killed with mechanical cervical dislocation (MCD) presented with more than one dislocation site. For average displacement distance, method (P = 0.003), age (P = 0.003), and method ^*^age (P = 0.489) were evaluated. For number of birds presenting with fractures, method ^*^age (P = 0.010 for age within MCD; P = 0.047 for the method at three weeks of age).

a, b *Indicates age differences within a method*.

c, d *Indicates method difference within an age group*.

No difference was observed between aCD and CD for displacement distance (*P* = 0.521). An effect of the method was observed for the average displacement distance with a greater distance observed in birds killed with CD compared to MCD (7.0 ± 1.0 mm vs. 2.0 ± 0.6 mm; *P* = 0.003). An age effect was observed with a greater distance observed in 3-week old turkeys compared to 1-week old poults (6.0 ± 1.0 mm vs. 2.0 ± 0.8 mm; *P* = 0.003).

No difference was observed between aCD and CD for the number of birds with fractures (*P* = 0.247). A method by age interaction was observed between CD and MCD for the number of birds with fractures, with greater numbers present in 3-week old turkeys killed with MCD compared to 1-week old poults (7/9 vs. 0/5; *P* = 0.010). Additionally, a greater number of fractures was observed in 3-week old turkeys killed with MCD compared to CD (7 of 9 vs. 4 of 9; *P* = 0.047). Multiple fracture locations were observed in 3-week old birds (n = 7) that presented with dislocation sites below the first cervical vertebrae. All fractures were in either C1 or C2 for turkeys killed by CD; for those killed with MCD, 33% occurred in C2 and 67% occurred in C3.

### Macroscopic and microscopic assessments of cerebrospinal trauma

Macroscopic scores were first compared between tissues from birds killed by aCD and CD. No effects of anesthesia were observed for any measure and therefore data from these birds were combined for comparison between CD and MCD for 3-week old turkeys. Only 1 of 18 birds killed by CD presented with a laceration to the neck whereas 5 of 9 birds killed by MCD presented with a laceration and/or external hemorrhage (*P* = 0.002). Turkeys in all treatments presented with moderate to marked subcutaneous hemorrhage of the head and neck. Spinal cord transection was not assessed in the five 1-week old poults because they were killed by manual cervical dislocation. Spinal cords were noted to be transected for 8 of 10 birds killed by CD at 1 week of age, 14 of 17 killed by CD at 3 weeks of age, and 9 of 9 killed by MCD at 3 weeks of age.

There were no microscopic differences in cerebrospinal tissue scores from birds killed by aCD or CD and CD results were combined and compared to MCD (Table 5). There were no differences between CD and MCD in the brain parenchymal (*P* = 0.627), spinal subdural (*P* = 0.402), or spinal parenchymal (*P* = 0.102) scores. Overall, turkeys killed using CD had higher scores for subdural brain hemorrhage (*P* = 0.020). Of the 18 birds sampled for CD, 9 had scores of 2 or greater indicating mild (5–10%) to marked (>30%) tissue sample damage, whereas cerebrospinal tissues from 5 of 5 birds killed with MCD had microscopic scores of 0 or 1.

**Table 5:** Results of the brain and spinal cord histology scores following application of killing methods.

	**Sample size**	Score^*^	**0**	**1**	**2**	**3**	**4**	**Anesthesia*^a^***	**Method *^b^***
Subdural (Brain)	8	CD	3	0	2	2	1	0.489	**0.020**
	10	aCD	3	3	3	1	0		
	5	MCD	3	2	0	0	0		
Parenchymal (Brain)	8	CD	7	1	0	0	0	0.975	0.627
	10	aCD	6	3	1	0	0		
	5	MCD	4	1	0	0	0		
Subdural (Spinal cord)	8	CD	5	2	0	1	0	0.175	0.402
	8	aCD	6	1	0	1	0		
	4	MCD	1	0	2	1	0		
Parenchymal (Spinal cord)	8	CD	4	3	0	1	0	0.218	0.102
	8	aCD	5	1	1	0	1		
	4	MCD	0	0	2	2	0		

* *Number of birds assigned each hemorrhage score (0–4)*.

a *The effect of anesthesia compares the changes noted in birds anesthetized and killed by manual cervical dislocation (aCD) vs. changes in awake birds killed by manual cervical dislocation (CD)*.

b *If the effect of anesthesia was non-significant, CD (anesthetized and awake combined) was compared to mechanical cervical dislocation (MCD); if the effect of anesthesia was significant only anesthetized CD was compared to MCD. Two brains (from CD) and three spinal cord sections (two from CD and one from MCD) were not included because they were lost during sample preparation and process*.

*Bold indicates significant values*.

## Discussion

The anesthesia protocol used in this study allowed for critical brainstem reflexes and post-mortem scores to be assessed while eliminating or minimizing potential pain and distress experienced by the turkeys. All birds were deemed to be generally unresponsive and showed minimal or no pedal withdrawal reflex prior to application of the killing methods. Since various agents used for anesthesia can affect some reflexes differently (12), it was important to first compare CD to aCD to determine how measures from anesthetized birds compared and could be interpreted for assessment of a previously untested killing method. Three ante-mortem measures were affected by the anesthesia protocol: the presence of the nictitating membrane reflex, the presence of clonic convulsions, and duration of gasping.

The presence of the nictitating membrane reflex was greatly reduced or not present at all as a result of anesthesia. This was contrary to the findings of Sandercock and colleagues (11) in which the nictitating membrane reflex persisted until after respiratory arrest and brain death in laying hens and turkeys anesthetized with sevoflurane and suggests that nictitating membrane reflex is more sensitive to differences between agents. The incidence of clonic convulsions was 100% for awake turkeys killed with CD but was reduced in turkeys that were anesthetized. Convulsions have been used in previous studies as approximate measures of brain death when other measures are not practical on-farm (9, 10, 18, 19). However, our results indicate that clonic convulsions was reduced in anesthetized birds and may not be useful as an approximate measure of brain death in cases where an anesthetic agent has been administered. The agents used in our study also increased the duration of gasping.

The primary objective of this study was to assess the efficacy of CD and MCD on poults and young turkeys. Pupillary eye reflex (an indicator of brain death) was observed in all birds for some time following application of either killing method. This is comparable to previous research that showed eye reflexes persisted for ~40 s in poultry successfully killed with manual CD (10, 20) but longer than the duration following application of another design of MCD (burdizzo) (10). Gregory and Wotton (6) found that birds killed with CD and an MCD (Semark neck pliers) exhibited visual evoked responses (EEGs) with shorter time to loss of visual evoked responses with CD compared to MCD. Jaw tone was also measured in our study because it was previously found to be a reliable measure used to differentiate between awake and insensible states during anesthesia in turkeys (11). Jaw tone was present with a combined incidence exceeding 80% of anesthetized birds, confirming that birds were sedated or within a light plane of anesthesia. Assuming that jaw tone can be used as an indicator of complete loss of sensibility, the presence and persistence of jaw tone in our study suggest that a high proportion of birds remained sensible following the application of both methods.

The MCD used in our study was not effective for killing 1-week old poults since the method failed to abolish the pupillary eye reflex or cause cardiac arrest in 100% of the birds within 5 min. After five unsuccessful attempts of the MCD device on 1-week old poults, no additional birds were tested with this device. This result differs from other studies that have demonstrated high kill rates, despite the presence of eye reflexes, using other styles of MCD [neck pliers (6); Burdizzo (10); novel glove with metal inserts, (20)], although none of the aforementioned studies used poults. It should be noted that MCD devices vary in their design and application and so conclusions about one type of MCD cannot be generalized to another MCD.

Although the KED-s was ineffective for poults, it was effective for killing 3-week old turkeys when allowing a 5-min cut-off period to reach brain death. The method success in 3-week old turkeys was consistent with the kill rates in other studies using MCD in older birds [neck pliers (6); Burdizzo (10); novel glove with metal inserts, (20)]. Ante-mortem measures were confirmed through the analysis of radiographs whereby images from 1-week old poults indicated no dislocations. Despite the specific design of the KED-s for killing small poultry, the 1-week old poults appeared to be too small for the device to cause cervical dislocation. For all other birds, radiographic assessment indicated that neither method resulted in a high degree of accuracy or precision. However, compared to MCD, CD resulted in more dislocations of the cervical vertebrae between C0 and C1, with a greater displacement distance between vertebrae. In 3-week old birds, MCD resulted in all dislocations lower along the cervical vertebrae with the greatest percentage between C2-C3. CD also resulted in birds presenting fewer fractures than MCD. The dislocation varied from previous research with manual CD or with an alternative method of MCD, in which 76 of the 85 (89%) chickens and turkeys (>3 kg) presented with cervical dislocation between the skull and C1 (21). The location of the fractures followed the same trend as the dislocation sites, with fractures occurring higher along the cervical vertebrae with CD compared to MCD. Bader et al. (21) found that fractures occurred at the first cervical vertebra in 36 of the 44 birds presenting with fractures. In addition to comminuted and transverse fractures, which were present in both CD and MCD, fragmentation occurred solely in birds killed with MCD. Differences in dislocation site and fractures to previous research with turkeys may be due to differences in device design, bird age, and size, or operator experience in applying the device.

In the 3-week old birds, MCD did cause dislocation of the vertebrae and transection of the spinal cord, but the displacement location was more variable with more fractures noted compared to CD. As outlined in the AVMA (4) guidelines, the primary crushing of the cervical vertebrae or spinal cord is unacceptable unless first rendering the animal insensible. As presented, it is clear that fractures were present with both methods; however, MCD resulted in a higher incidence of fractures. The number of birds (13 of the 42 total or 31%) presenting with fractures in the present study was lower than the 52% (44 of the 85 birds) of birds presenting with fractures in a study by Bader et al. (21). That study did not distinguish between fractures occurring as a result of manual vs. mechanical cervical dislocation.

Overall, few differences were found for macroscopic or microscopic hemorrhage and trauma scores between CD and MCD, with the exceptions of external hemorrhage and skin laceration and microscopic subdural brain hemorrhage. This was unexpected since CD was anticipated to produce more damage as a result of the pulling, twisting, and other physical trauma that results from stretching the neck. However, sample sizes were small and unbalanced. The small sample size of brains and spinal cords for microscopic scoring prevented method by age interactions to be compared and also limited the power of the statistical tests. Overall, there was some macroscopic damage present in all birds with minimal to no microscopic damage to either the brain or spinal tissue. This result was consistent with a previous study where macroscopic hemorrhage was present in all turkeys, but the microscopic damage was only present in 1 of 4 turkeys killed with MCD (Burdizzo) and 1 of 4 turkeys killed with cervical dislocation (22). Gregory and Wotton (6) suggested that a concussive force is required to ensure immediate insensibility following cervical dislocation with resulting death by ischemia. Only 4 of 18 turkeys killed with CD in this study showed moderate to severe microscopic subdural brain hemorrhage, and the ante-mortem measures suggest that birds killed by either CD or MCD did not experience sufficient damage to the brain to induce immediate loss of jaw tone (indicator of complete loss of sensibility) or pupillary light reflex (indicator of brain death). We hypothesize that death was likely achieved by anoxia for both killing methods, regardless of turkey age.

Limitations of this study include that operators were not randomly assigned to methods, the sample size was limited, and reflex and behavioral measures were recorded by observer unblinded to treatment. However, macroscopic, radiographic, and microscopic assessments were conducted by individuals blinded to treatment and supported findings of unblinded reflex and behavioral measures.

In conclusion, the MCD was unsuccessful at killing 1-week old poults. For manual CD at both ages and MCD at 3 weeks of age, 100% of turkeys showed positive brainstem reflexes following method application, with prolonged duration of pupillary light reflex and jaw tone seen in birds killed by MCD. Radiographs indicated that MCD caused less displacement and more fractures compared to CD. In addition, the majority of turkey poults killed with either method had minimal microscopic and macroscopic hemorrhage suggesting that neither method caused a concussive force great enough to induce severe brain damage.

## Author contributions

TW, ST, PT, and KS-L are co-principal investigators and conceived and supervised the study. The experimental procedures were designed and performed by CW. PT developed a scoring method and interpreted all microscopic samples. HC developed the scoring method and interpreted the radiographs. LL was the attending clinical veterinarian and developed the anesthesia protocol. CW conducted all statistical analysis and wrote the paper. All authors edited the paper.

### Conflict of interest statement

TW is the Egg Farmers of Canada Chair in Poultry Welfare Research. The founding sponsors had no role in the design of the study; in the collection, analyses, or interpretation of data; in the writing of the manuscript, and in the decision to publish the results. The remaining authors declare that the research was conducted in the absence of any commercial or financial relationships that could be construed as a potential conflict of interest.
